# A Stand-Alone Microfluidic Chip for Long-Term Cell Culture

**DOI:** 10.3390/mi14010207

**Published:** 2023-01-14

**Authors:** Yibo Feng, Yang Zeng, Jiahao Fu, Bingchen Che, Guangyin Jing, Yonggang Liu, Dan Sun, Ce Zhang

**Affiliations:** 1 State Key Laboratory of Photon-Technology in Western China Energy, Institute of Photonics and Photon-Technology, Northwest University, No. 1, Xuefu Avenue, Xi’an 710127, China; 2 School of Physics, Northwest University, No. 1 Xuefu Avenue, Xi’an 710127, China; 3 Laboratory of Stem Cell and Tissue Engineering, Chongqing Medical University, Chongqing 400016, China; 4 RongGuangYun Biotechnology Co., Ltd., No. G2018, Building C, Qin Han Innovation Center, Xianyang 712039, China

**Keywords:** cell culture, fluidic chip, ITO glass, in-field, live cell

## Abstract

Live-cell microscopy is crucial for biomedical studies and clinical tests. The technique is, however, limited to few laboratories due to its high cost and bulky size of the necessary culture equipment. In this study, we propose a portable microfluidic-cell-culture system, which is merely 15 cm×11 cm×9 cm in dimension, powered by a conventional alkali battery and costs less than USD 20. For long-term cell culture, a fresh culture medium exposed to 5% CO_2_ is programmed to be delivered to the culture chamber at defined time intervals. The 37 °C culture temperature is maintained by timely electrifying the ITO glass slide underneath the culture chamber. Our results demonstrate that 3T3 fibroblasts, HepG2 cells, MB-231 cells and tumor spheroids can be well-maintained for more than 48 h on top of the microscope stage and show physical characters (e.g., morphology and mobility) and growth rate on par with the commercial stage-top incubator and the widely adopted CO_2_ incubator. The proposed portable cell culture device is, therefore, suitable for simple live-cell studies in the lab and cell experiments in the field when samples cannot be shipped.

## 1. Introduction

Live-cell techniques in vitro are crucial for biological and biomedical studies, e.g., the dynamics of tissue development and cancer metastasis [1,2,3]. Since 1665, when Robert Hooke discovered the structure of cells [4,5], it has increasingly been appreciated that the collective behavior of individual and population cells plays crucial roles in regulating life machinery [6]. Currently, live-cell platforms, e.g., live-cell microscopy [7,8,9,10] and organ on chip [11,12,13,14], are, however, limited to laboratory use due to the large volume and complicated pipelines of the culture equipment.

To overcome the above-mentioned difficulties, we herein propose a portable cell culture device, which can replace the function of traditional culture equipment, similar to a mobile phone in dimension and costs merely USD 20 to build. Our device consists of a cell-culture chip, where cells can be maintained, and a control unit, controlling the temperature and timely delivery of a fresh culture medium. Although some reports on microfluidic-cell-culture devices have their own characteristics [15,16,17], these devices can only be used for cell culture and cannot control the culture environment. These devices are equivalent to the functions of the cell-culture chip unit of our device, and they must be used together with traditional cell culture equipment (e.g., the cage incubator from OKO Labs). Our device can independently in-field cell culture for a long time without cell culture equipment.

Our device was powered by an alkali battery, and culture conditions can be well-maintained for more than 48 h without recharging. To demonstrate the viability of different cell types in the fluidic device, e.g., the cell growth rate and migratory behavior, all experiments were performed on top of a microscope stage. The results showed that cell lines, including 3T3, HepG2, MB-231 and tumor spheroids composed of 3T3 and HepG2, showed mobility and a proliferation rate comparable to a conventional CO_2_ incubator. Besides live-cell studies in the lab, these results are also important for performing live-cell studies in the field, such as tests on sperm and foodborne pathogens [18,19]. Some of the main reasons are listed as follows: the semen quality assessment has to be completed within a few hours to ensure the sample is being tested in the optimal condition [18]; the in-field testing of food is mandatory for the detection of foodborne pathogens [19]; to detect infectious biomarkers in clinical samples, many laws have strict requirements on the release of living organisms, so immediate detection and destruction outside the laboratory or the transfer of living organisms in closed devices is necessary [20]; and due to the harsh conditions in remote areas, where biological samples have to be stored and transported, a point-of-care test (POCT) device maintaining culture conditions is also necessary [21,22]. These indicate broad applications in the field of biological and biomedical research, as well as in-field assays.

## 2. Materials and Methods

### 2.1. Design and Fabrication of Chip

The fluidic device contains 2 parts: a cell-culture chip and a control unit. The fluidic chip consists of 4 layers, which are the light shield, the culture chamber, the ITO glass and the frame holding pipelines on the order of assembly. The overall size of the culture chamber is 29 mm × 26 mm × 14 mm. The center is composed of three hollowed-out structures; namely, the upper and lower parts are cylinders with a radius of 8 mm and a height of 6 mm, and a radius of 8 mm and a height of 5 mm, and the middle part is connected by a cylinder with a radius of 5 mm and a height of 3 mm. The exchange of liquid is carried out in the superstructure. The liquid inlet hole and the liquid outlet hole are on both sides of the upper cylinder, with a diameter of 1.6 mm. The liquid inlet hole is 4.8 mm from the top of the upper layer, and the liquid outlet hole is 1.5 mm from the top of the upper layer. When the culture solution is input, the culture solution will flow to the grid between the upper and middle layers first. By adjusting the flow rate, the input culture solution can generate reflux in the round cavity.

The control unit is composed of a peristaltic pump, whose operating frequency is controlled by a medium-exchange board; a water tank housing the culture medium; and an alkali battery, powering all the electronic units and ITO glass. The cover of the control unit, light shield and frame of the fluidic chip was designed using Solidworks software (Premium 2018 sp5.0, Dassault Systèmes Solidworks Corporation, Paris, France) and produced using a 3D printer (CR-3040, CREALITY, Shenzhen, China). The water tank in the control unit was made using a light-curing printer (Lite600, UnionTech, Shanghai, China), and the acrylic cultivation chamber was made in a processing factory by laser engraving after we designed the drawings.

To assemble the fluidic chip, a thin layer of PDMS (Polydimethylsiloxane, 10:1 of monomer: catalyst ratio) was firstly spin-coated on top of ITO glass at 1800 rpm, immediately after which the acrylic chamber was placed on the PDMS for bonding. After curing for 24 h at 40 °C, the culture chamber was mounted on the 3D-printed chip frame together with pipelines, electrodes and a temperature sensor. The light shield was positioned only after cell loading. A similar procedure was applied when assembling the control unit.

### 2.2. Chip Control and Operation 

The operation procedure of the fluidic device is shown in Figure S1. In brief, the fluidic chip was firstly connected to the control unit, including the pipeline and electric circuit, following which the culture medium was placed into the water tank. The power of the control unit was then turned on, and the temperature in the fluidic chip was set to be 37 °C. The medium exchange between the water tank and the culture chamber ranged between 1 min^−1^ and 1 h^−1^ depending on the cultured cell type and cell number. The upper chamber of the water tank was continuously filled with 5% CO_2_ to prevent changes in medium pH caused by cell metabolism. Target cells could then be loaded into the culture chamber, which was sealed with PCR tape.

### 2.3. Cell Culture

3T3 mouse fibroblasts, MB cells and HepG2 cells were routinely cultured in culture flasks. The medium used in this paper consisted of Dulbecco’s modified Eagle medium (DMEM, Gibco, Brooklyn, NY, USA) with 10% fetal bovine serum (FBS, Gibco, Brooklyn, NY, USA) and 1% penicillin–streptomycin solution (Invitrogen, Carlsbad, CA, USA). When the density exceeded 80% confluency, cells were digested using trypsin. The cell suspension was centrifuged and diluted with 1 mL of culture medium, and then 10 μL of cell suspension was collected and diluted to 1 mL in the culture medium before loading into the chip. Before cell loading, the bottom of the culture chamber was treated with fibronectin (0.25 mg/mL; Millipore, Wien, Austria) for 2 h.

The liver spheroid composed of 3T3 fibroblasts and HepG2 cells was formed by mixing and culturing these 2 cell types in a hanging droplet for 48 h at a ratio of 1 to 10. For 3D culture, 200 μL of collagen solution was deposited into the culture chamber and solidified before the culture medium.

### 2.4. Cell Proliferation and Statistical Analysis

A Nikon Ti2-ECLIPSE microscope with an automated translational stage and a digital CMOS camera (ORCA-Flash 4.0, Hamamatsu, Shizuoka, Japan) and a microscope (XDS-5, Bingyu, Shanghai, China) were used for cell image acquisition. Image acquisition was controlled by the Nikon microscope’s software (NIS components) and a custom-written control program, respectively. The position of individual cells (i.e., the x and y coordinates) was determined using a customized Matlab program and Image-J software. The cell number could, therefore, be automatically counted. We performed a minimum of three repetitions for each experimental condition (i.e., the cell types). Data are expressed as means ± SD.

### 2.5. Numerical Simulation

In numerical simulation, we firstly set up the cross-section of the culture chamber in COMSOL software. A multi-physical coupling interface between laminar flow and dilute matter transfer was selected for simulation. The initial concentration of the reagent in cell culture chamber was set to be zero. The input concentration of the matter at the inlet was set to be 1 mol/L.

## 3. Results

### 3.1. Parameter Determination of the Fluidic-Culture Device

**Figure 1:**
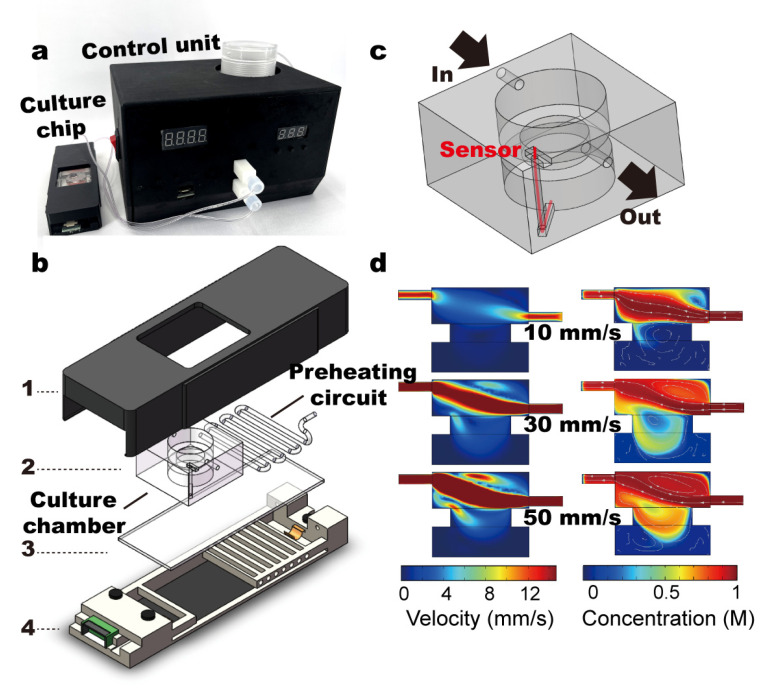
Design of fluidic-culture chip. (**a**). The cell culture device is composed of a fluidic-culture chip and a control unit. (**b**). The fluidic-culture chip was produced by stacking of (1) light shield, (2) PMMA-made culture chamber and pipelines, (3) ITO glass, (4) the electrodes and frame of chip. (**c**). Geometry of the culture chamber shows 2 parts separated by a narrow neck, i.e., the medium-exchange upper part and the cell-culture lower part. (**d**). Fluid distribution of different input velocities at 10 s. Numerical simulation (COMSOL) shows that bottom of the culture chamber was not affected by shear flow during medium exchange at input flow velocity up to 50 mm/s.

As is shown in (Figure 1a), the culture device consists of a cell-culture chip, where cells and tumor spheroids can be maintained, and a control unit, controlling the temperature and timely delivery of the fresh culture medium. The cell-culture chip is composed of an ITO-coated (indium tin oxide) glass substrate, which is connected to the control unit by two copper electrodes (Figure 1b); the PMMA-made culture chamber is separated from the ITO glass by a spin-coated PDMS layer to avoid direct contact between cells and the conductive metal layer (Figure 1b); pipelines (Cole-Parmer, 06419-01, Tygon, Malvern, PA, USA) are fixed on top of the substrate, so that the delivered culture medium can be preheated before entering the culture chamber (i.e., the preheating circuit) (Figure 1b); a thermal sensor is positioned at the bottom of the culture chamber (Figure 1c); and the whole chip is covered by a dark cover to eliminate light exposure (Figure 1b). To prevent unwanted shear, the bottom of the culture chamber is isolated from the medium-exchange part by a narrow neck (Figure 1c,d). In the numerical simulation, the initial concentration of reagent in cell culture chamber was set to be zero. The input concentration from the inlet was 1 mol/L. The simulated input flow velocity was 10 mm/s, 30 mm/s and 50 mm/s. Our results demonstrate that within 10 s (Video S1), ~50% of the culture chamber was filled with fresh culture medium at 10 mm/s input flow velocity and ~73% at 50 mm/s (Figure 1d). The flow velocity at the bottom of culture chamber remained zero at all times and thus brought no shear on the cells. Even though it took more than 48 h for the reagent concentration to reach equilibrium in the culture chamber (Video S3), the hourly medium exchange in our experiment delivered enough nutrients to maintain cells and tumor spheroids in the chip (Video S2). Due to the need for temperature control, our fluidic device uses an ITO-coated glass substrate. In this device, we used ITO glass whose length, width and thickness were 76 mm × 26 mm × 1 mm, and its resistance was 7~10 Ω.

**Figure 2:**
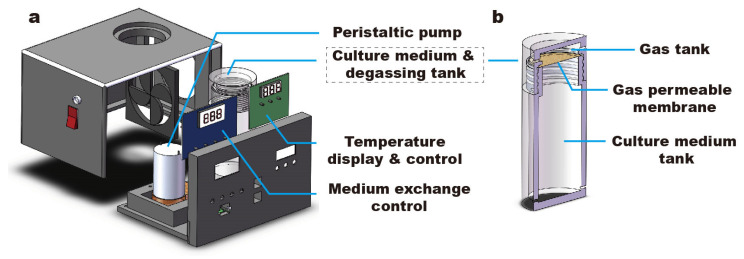
Design of control unit. (**a**). The control unit. (**b**). The water tank of control unit, in which the gas tank and the culture medium tank are separated by a gas-permeable membrane.

The control unit of the culture device was built by integrating a peristaltic pump (AQB-YS-12-G01, Cinyar, Shanghai, China), an alkali battery and electronic circuits controlling the on–off of the pump and electrifying the ITO glass (Figure 2a). For long-term culture, the temperature was maintained at 37 ± 0.1 °C by timely electrifying the underside of the ITO glass. We monitored the temperature change by positioning a sensor at the center of the culture chamber. We observed that it took approximately 2 min for the temperature to rise from room temperature (25 °C) to 37 °C and stabilize at 37 ± 3 °C (Figure S4). To avoid toxins and variations in the chemical environment (e.g., pH) caused by cell metabolism and to provide nutrients, the medium exchange was performed hourly. In the process of cell culture, 5% CO_2_ was continuously introduced into the liquid storage tank at a pressure lower than 1 psi. Separated by a Polytetrafluoroethylene microporous membrane with pore diameter of 1.2 µm, the medium was constantly exposed to 5% CO_2_ during culture (Figure 2b).

### 3.2. Performance of the Fluidic-Cell-Culture Device

**Figure 3:**
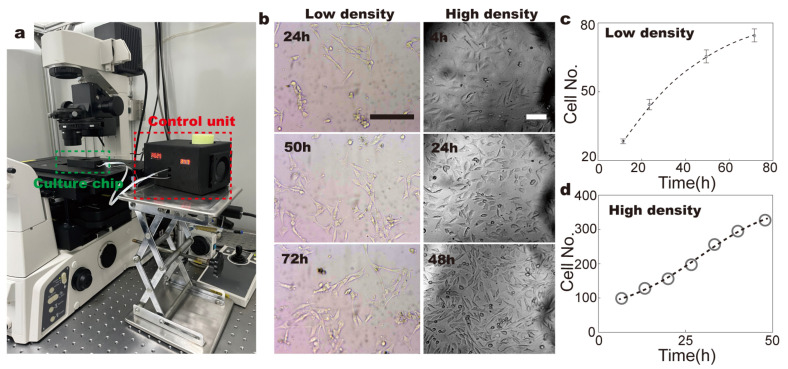
Application of the fluidic-culture chip in live-cell imaging. (**a**). Application of fluidic-culture chip in Inverted Fluorescence Microscope. (**b**). Cell line 3T3 fibroblasts at different seeding densities were cultured on chip for more than 48 h; the field of view is 340 μm × 250 μm. (**c**). Counts of 3T3 fibroblasts within 72 h, when being seeded at low cell density. (**d**). Proliferation of 3T3 fibroblasts at high seeding-cell density during 48 h culture on chip. Scale bars denote 100 µm in all figures. Dots are experimental data, and the dashed line is the fitting curve.

**Figure 4:**
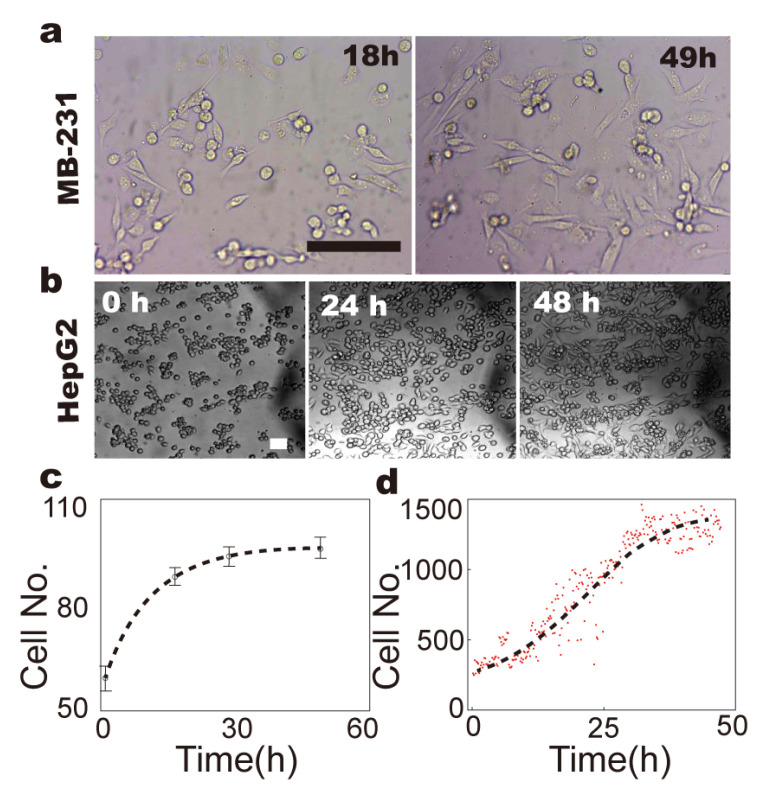
Culture of tumor-origin cells. (**a**,**b**). Live-cell images of MB-231 (**a**) and HepG2 cells (**b**) during 48 h culture on chip. (**c**,**d**). Growth rate of MB-231 (**c**) and HepG2 cells (**d**). Dots are experimental data, and the dashed line is the fitting curve. Scale bars denote 100 µm.

To monitor in real time the conformation, mobility and proliferation of different cell lines, including 3T3, HepG2 and MB-231 cells, the fluidic chip was mounted on top of a microscope stage (Figure 3a). Our results demonstrate that with hourly medium exchange and constant 5% CO_2_ exposure, the number of 3T3 fibroblasts nearly tripled within 48 h of incubation on the chip at both high and low seeding-cell density (0.05/cm^2^ and 0.02/cm^2^, respectively) (Figure 3b–d), which is comparable to the growth rate in a conventional CO_2_ incubator (Figure S5) [23,24,25]. A similar growth rate, which is reflected by the cell number and counts of cell division, was observed for tumor-origin cell lines, i.e., MB and HepG2 cells (Figure 4). These results indicate that the culture conditions were well-maintained in the fluidic-culture chip.

**Figure 5:**
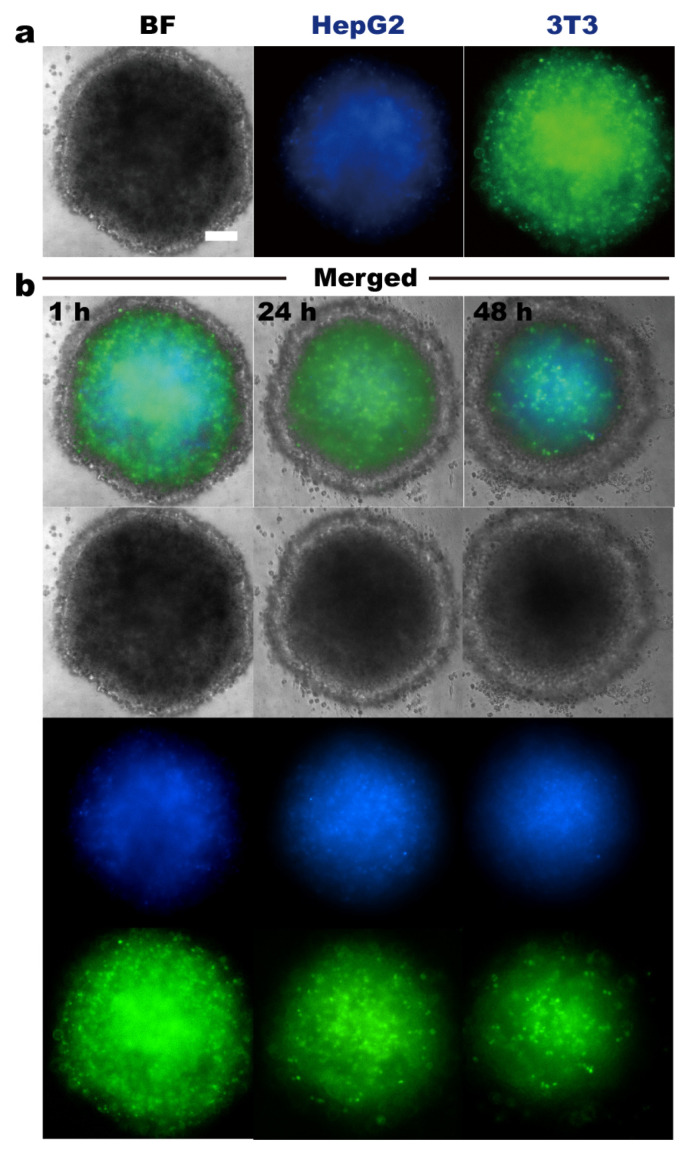
Culture of tumor spheroid formed by self-organization of 3T3 and HepG2 cells. (**a**). Live-cell images of tumor spheroid; nucleus of HepG2 cells was stained by DAPI, and 3T3 fibroblasts by H2B-FITC. (**b**). Conformational changes in tumor spheroid during 48 h culture on the chip. Scale bar denotes 100 µm.

The fluidic-culture device is suitable for relatively large tissue samples (i.e., tumor spheroids of a few hundreds of μm) as well (Figure 5). Using a tumor spheroid, which is produced by depositing 3T3 and HepG2 cells in a hanging droplet [26], our results demonstrate that tumor spheroids maintained in the fluidic-culture chip show similar behavior to those in microscopic culture equipment. Liver tumor spheroids could be well-maintained for days, showing compact and round-shaped morphology. Fibroblasts self-organized into a protective fibroblast–collagen–matrix layer, and HepG2 cells rested at the core region (i.e., the cage incubator from OKO Labs, Figures S2 and S3) [26]. Firstly, 3T3 fibroblasts and HepG2 cells self-organized into layered structures with HepG2 cells in the core region. Secondly, with prolonged incubation, cells in the peripheral region of the spheroid started to adhere to the untreated PDMS substrate, reflected by the decreasing spheroid diameter and increasing width of the outer ring during 48 h culture on the chip (Figure 5b). As shown in Figure 5, HEPG2 cells were labeled with DAPI (blue) in the center, and 3T3 fibroblasts were stained by H2B-FITC (green). After 1 h of incubation, the diameter of the tumor spheroid was about 650 µm, the center was about 548 µm, and the edge was about 45 µm. After 24 h, the spheroid diameter changed to ~612.5 µm, the central region changed to ~475.5 µm, and the peripheral-region thickness changed to ~70.9 µm; after 48 h, the spheroid diameter changed to ~640 µm, the central region changed to ~423.5 µm, and the peripheral-region thickness changed to ~100 µm, respectively. Previous studies reveal that in many solid tumors, the ECM comprises up to 60% of the tumor mass, and it is secreted to a large degree by fibroblasts [27]. Therefore, it is plausible that under suitable culture conditions provided by the culture device, fibroblasts secrete ECM and promote both cell types to adhere to the substrate.

## 4. Conclusions

We herein propose a portable fluidic-culture device in which conditions suitable for long-term live-cell studies can be well-maintained. Our results demonstrate that the growth rate of 3T3 fibroblasts, MB and HepG2 cells in our fluidic-culture device is comparable to the growth rate in a conventional CO_2_ incubator. We also found it suitable for relatively large tissue samples. Considering its small dimension and low cost (Table S1), the device can be integrated with any platform (e.g., an inverted microscope or even a mobile camera) and thus shows great potential for in-field biomedical studies and clinical tests.

## Data Availability

Not applicable.

## Supplementary Materials

The following supporting information can be downloaded at: https://www.mdpi.com/article/10.3390/mi14010207/s1. Figure S1: Operation procedure of cell culture in the proposed fluid chip; Figure S2: Culture of liver tumor spheroids; Figure S3: 3D structure of liver tumor spheroid observed using z-stack fluorescence imaging; Figure S4: Temperature variation in the culture chamber; Figure S5: Comparison of proliferation of 3T3 cells in culture chip and incubator; Table S1: Costing list of the devices in the paper; Video S1: Simulate the distribution of substances in the culture chamber; Video S2: The diffusion of simulated substances in the culture chamber after 10s of introduction. Video S3: The diffusion of simulated substances in the culture chamber after 10s of introduction.
