# Supplementary material for: A Stand-Alone Microfluidic Chip for Long-Term Cell Culture
Source: Micromachines (Basel). 2023 Jan 14;14(1):207. doi: 10.3390/mi14010207 (PMC9863834; doi:10.3390/mi14010207)
Supplement: Supplementary file 1 [file micromachines-14-00207-s001.zip › Supplementary Information.pdf]

Supplementary Information

A stand-alone microfluidic chip for long-term cell culture

Yibo Feng [1,†], Yang Zeng [1,†], Jiahao Fu [1], Bingchen Che [2], Guangyin Jing [2], Yonggang Liu [3,*], Dan Sun [1,4,*] and Ce Zhang [1,4,*]

[1] State Key Laboratory of Photon-Technology in Western China Energy, Institute of Photonics and Photon-Technology, Northwest University, No. 1, Xuefu Avenue, Xi'an, 710127, Shaanxi, China

[2]School of physics, Northwest University, No. 1 Xuefu Avenue, Xi'an, 710127, Shaanxi, China

[3]Laboratory of Stem Cell and Tissue Engineering, Chongqing Medical University, Chongqing, 400016, China

[4]RongGuangYun Biotechnology Co., LTD, No. G2018, Building C, Qin Han Innovation Center, Xianyang, 712039, Shaanxi, China

This PDF file includes:

Figure S1 to S5

Table S1

Other supplementary materials for this manuscript include the following:
Video S1 to S3

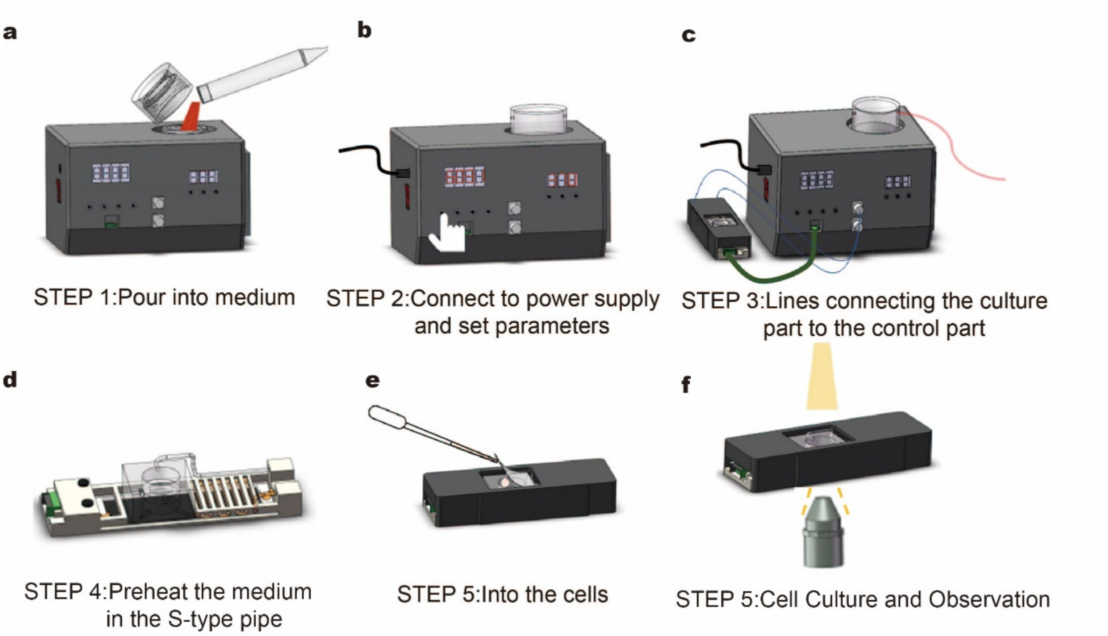

Figure S1. Operation procedure of cell culture in the proposed fluid chip. a. Open cover of the liquid storage tank of the device and pour the medium required for cell culture. b. Connect the power cable, turn on the device switch, and adjust the circulation time and interval of culture medium. c. Control lines and medium circulation lines connecting the control section to the culture section. d. Transport the medium to the S-shaped pipe in front of the acrylic chamber for preheating. e. Open the PCR membrane at the top of the culture chamber and seed into the cells. f. Device operation, cell or tissue culture, and state observation with the help of microscopic equipment at any time.

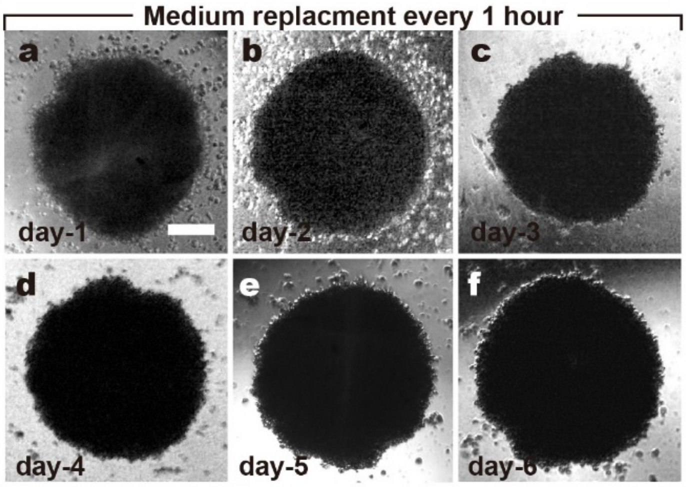

Figure S2. Culture of liver tumor spheroids. A pre-formed spheroid (24 h in hanging droplet) cultured for a to f 1 to 6 days [1].

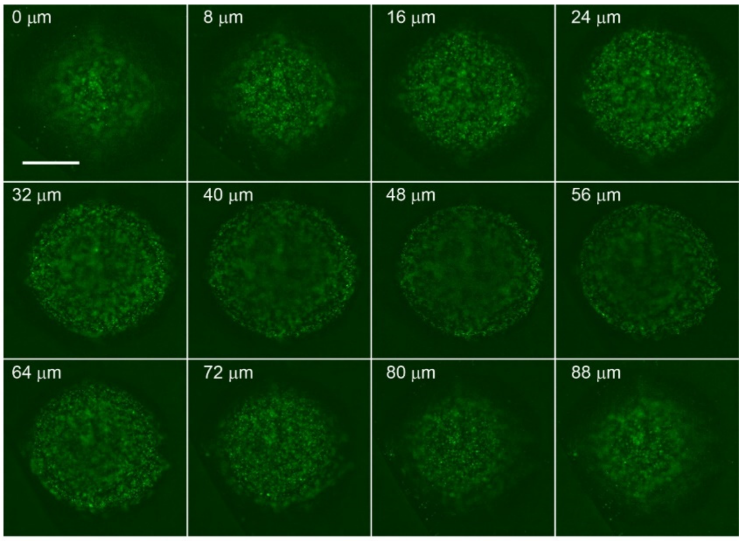

Figure S3. 3D structure of liver tumor spheroid observed using z-stack fluorescence imaging. We observe that fibroblasts distribute mostly at the outer layer of the spheroid. Scale bar denote 300 mm [1].

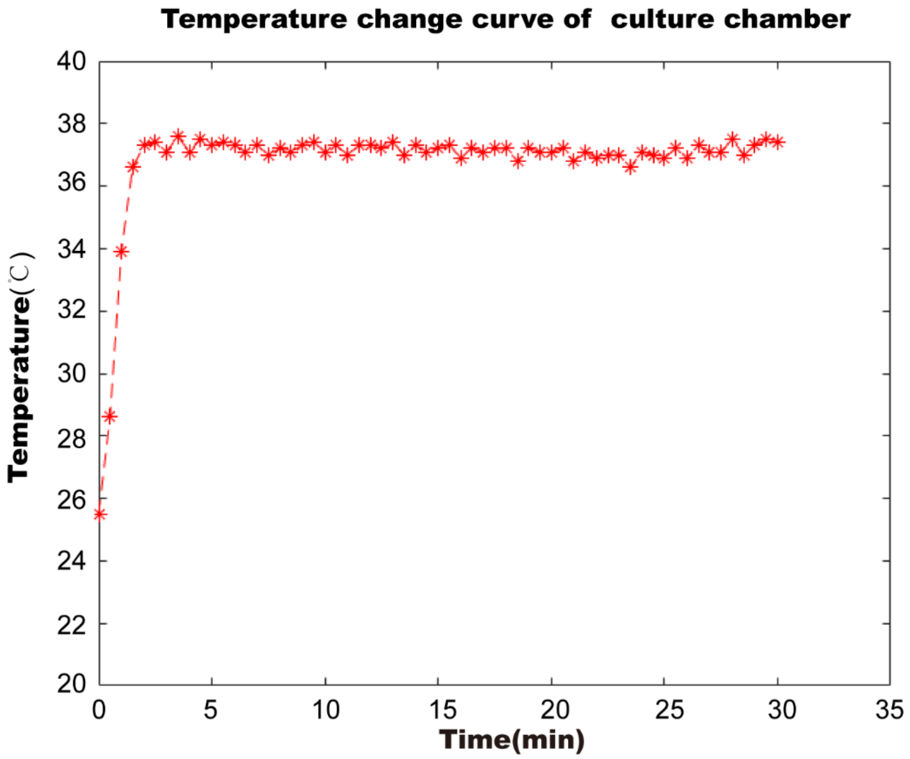

Figure S4. Temperature variation in the culture chamber.

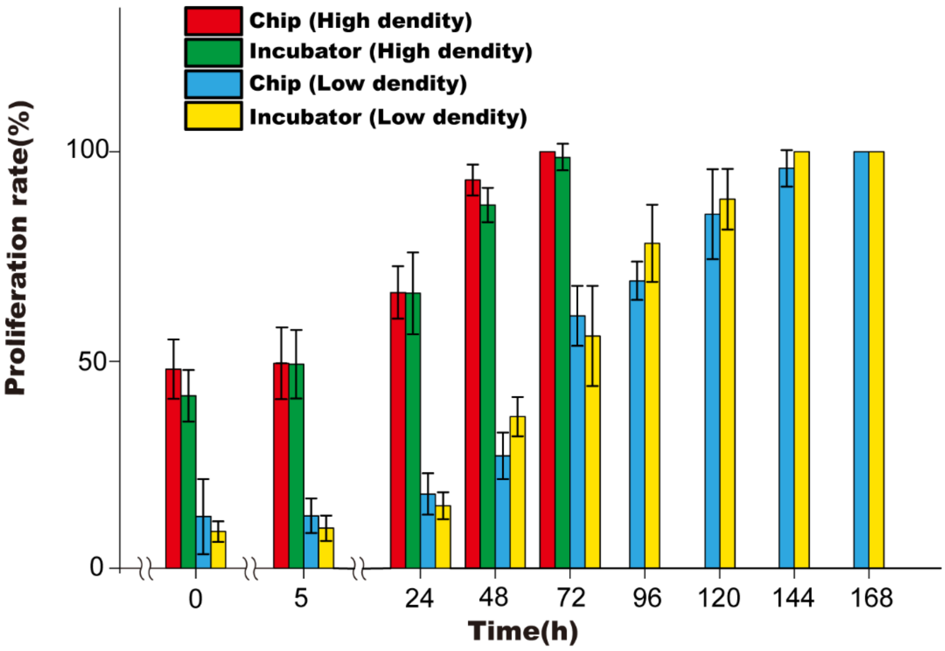

Figure S5. Comparison of proliferation of 3T3 cells in culture chip and incubator. The results in the figure show that the cell proliferation efficiency in the culture chip is roughly equal to that in the incubator. At the same time, under the initial condition of low dendity, the cells can be cultured for more than one week.

Table S1: Costing list of the devices in the paper

Ito glass	Acrylic chip	Connectors, wires, etc	Pipeline	Temperature controller	Gasket	Time controller	Peristaltic pump	Thermistor	SUM
10 ¥	45 ¥	1.8 ¥	12.3 ¥	5 ¥	0.34 ¥	18.8¥	43.65¥	0.3 ¥	$19.673

Video S1. Simulate the distribution of substances in the culture chamber. The inlet velocity of the video

is 50 mm/s, the initial concentration of the material is 1 mol/L, and the diffusion coefficient is 1×10^{-11} m^2/s. During the 10 s, the material gradually fills the cavity, while keeping a distance from the bottom of the cavity.

Video S2. The diffusion of simulated substances in the culture chamber after 10 s of introduction. The diffusion coefficient is set to 1×10^{-11} m^2/s. In the process of 2 hours, the substance gradually spread out, and the initial substance concentration at the bottom of the cavity was 0 mol/m^2. After 2 hours, the bottom could reach 0.228 mol/m^2.

Video S3. The diffusion of simulated substances in the culture chamber after 10 s of introduction. The diffusion coefficient is set to 1×10^{-11} m^2/s. After 24 hours, the medium exchange in the culture chamber tends to be balanced.

References

1. Feng, Y.; Wang, B.; Tian, Y.; Chen, H.; Liu, Y.; Fan, H.; Wang, K.; Zhang, C. Active fluidic chip produced using 3D-printing for combinatorial therapeutic screening on liver tumor spheroid. *Biosens. Bioelectron.* **2020**, *151*, 111966.
